# Protein Kinase CK2 and Epstein–Barr Virus

**DOI:** 10.3390/biomedicines11020358

**Published:** 2023-01-26

**Authors:** Mathias Montenarh, Friedrich A. Grässer, Claudia Götz

**Affiliations:** 1 Medical Biochemistry and Molecular Biology, Saarland University, Buildings 44 and 47, 66424 Homburg, Germany; 2 Institute of Virology, Saarland University, Buildings 44 and 47, 66424 Homburg, Germany

**Keywords:** protein kinase CK2, phosphorylation, Epstein–Barr virus, EBV-encoded proteins, signaling pathways, p53, review

## Abstract

Protein kinase CK2 is a pleiotropic protein kinase, which phosphorylates a number of cellular and viral proteins. Thereby, this kinase is implicated in the regulation of cellular signaling, controlling of cell proliferation, apoptosis, angiogenesis, immune response, migration and invasion. In general, viruses use host signaling mechanisms for the replication of their genome as well as for cell transformation leading to cancer. Therefore, it is not surprising that CK2 also plays a role in controlling viral infection and the generation of cancer cells. Epstein–Barr virus (EBV) lytically infects epithelial cells of the oropharynx and B cells. These latently infected B cells subsequently become resting memory B cells when passing the germinal center. Importantly, EBV is responsible for the generation of tumors such as Burkitt’s lymphoma. EBV was one of the first human viruses, which was connected to CK2 in the early nineties of the last century. The present review shows that protein kinase CK2 phosphorylates EBV encoded proteins as well as cellular proteins, which are implicated in the lytic and persistent infection and in EBV-induced neoplastic transformation. EBV-encoded and CK2-phosphorylated proteins together with CK2-phosphorylated cellular signaling proteins have the potential to provide efficient virus replication and cell transformation. Since there are powerful inhibitors known for CK2 kinase activity, CK2 might become an attractive target for the inhibition of EBV replication and cell transformation.

## 1. Introduction

Protein phosphorylation is an essential post-translational modification of proteins in most cellular processes, and also in viral replication as well as in virally induced neoplastic transformation. This post-translational modification is achieved by about 518 different protein kinases expressed in eukaryotic cells [1]. Among these kinases, protein kinase CK2 (formerly known as casein kinase 2) is responsible for about 25% of the cellular phosphoproteome [2]. CK2 is a ubiquitously expressed serine/threonine protein kinase composed of two catalytic subunits (α or its isoform α’) and two non-catalytic β subunits [3], which form the tetrameric holoenzyme. CK2α and CK2α’ are also active as kinases in the absence of CK2β [4,5]. CK2α and CK2α’ share some common functions but they also have unique functions [6,7,8,9]. The substrate specificity varies for the holoenzyme and the individual CK2α and CK2α’ subunits [8]. In addition to the protein kinase activity, the CK2 subunits bind to numerous cellular and viral proteins [10]. These protein–protein interactions are implicated in targeting CK2 subunits or the holoenzyme to specific target proteins in different cellular compartments [10,11]. The binding of CK2β to other protein kinases appears to play a role in the regulation of these kinases such as A-raf, c-mos, p90rsk (for review see: [12]), PKC [13] or CHK-1 [14]. CK2 is a regulator of the PI3K/Akt, NF-κB, Wnt/β-catenin and JAK/STAT signaling cascades [15,16,17,18,19,20]. CK2 phosphorylates serine or threonine residues in a consensus sequence S/T-x-x-E/D/pS/pT, where x can be any amino acid with the exception of proline [21]. This consensus sequence is often found in an acidic environment. CK2, which has more than 500 known substrates [22], phosphorylates and thereby modulates the activities of viral and cellular proteins [23]. CK2 substrates include proteins involved in the regulation of gene expression or protein synthesis. Other substrates are implicated in cell growth, proliferation, survival or metabolic processes [22,24,25]. CK2 is associated with a high proliferation rate. It is therefore not surprising that this kinase also plays a role in the formation of virally induced tumors. Moreover, CK2 expression and protein kinase activity are higher in a variety of solid tumors compared to the normal tissue or cells [26]. The first experiments to demonstrate an oncogenic potential of CK2 date back to 1995, when Seldin and Leder showed that overexpression of the catalytic subunit of CK2 together with myc was capable of transforming lymphocytes [27]. This property of CK2 renders the kinase a suitable target for therapeutic treatment of tumor patients. Over the last decade, a great number of different inhibitors for the kinase activity of CK2 have been established [28,29,30,31]. Most of these inhibitors are ATP competitive inhibitors, although other inhibitors have also been introduced [32]. Comprehensive descriptions of CK2 inhibitors can be found in a number of reviews [30,33,34,35,36]. Recently, Wells et al. described a new CK2 inhibitor, which is highly CK2 specific and which did not influence the proliferation of cancer cells [31]. The role of this new CK2 inhibitor in viral replication and virally induced cell transformation awaits further analysis. Initially, phosphorylation by CK2 was demonstrated for the human papilloma virus E7 protein [37]. Later, it was shown that CK2 plays a role in infectious diseases caused by adeno viruses [38], hepatitis C virus (HCV) [39], human cytomegalovirus (HCMV) [40], human immunodeficiency virus (HIV) [41], human T-lymphotropic virus type 1 (HTLV-1) [42], human papilloma virus (HPV) [43], herpes simplex-1 virus (HSV) [44], SARS-CoV-2 [45] and also by Epstein–Barr virus (EBV) [46].

## 2. Epstein–Barr Virus

It has been known for many years that viruses account for about 10–15% of all cancer cases world-wide [47,48]. Epstein–Barr virus (EBV) was first discovered in continuously growing tumor cells derived from patients with Burkitt’s lymphoma [49]. EBV infects B cells of the immune system and epithelial cells. After the initial lytic infection, most likely in oropharyngeal epithelial cells, EBV latently persists in memory B cells for the rest of the infected individual’s life [50,51]. After infection, the linear viral DNA circularizes due to the cellular repair mechanism that joins free DNA ends. The viral genome remains in the nucleus as a circular episome. EBV infection in early childhood mostly takes place sub-clinically. Infected B-cells, when passing the germinal center (GC), may convert into functional memory cells; when the memory is recalled by contact with antigen, the cells mature into plasma cells and thereby shed both antibodies and virus. The resting memory B-cells do not express EBV proteins but various non-coding miRNAs and the so-called EBER RNAs. This type of infection is called latency 0 [52]. In endemic Burkitt’s lymphoma, the viral nuclear antigen 1 (EBNA-1), the non-coding EBER1 and EBER2 RNAs, the so-called BART miRNAs as well as a viral snoRNA are expressed (latency I). In Hodgkin’s disease (HD), diffuse large B-cell lymphoma (DLBCL), nasal NKT- cell lymphoma (NKTL), nasopharyngeal carcinoma (NPC) and gastric cancer (GC), the latent membrane proteins LMP1, LMP2A and LMP2B are expressed in addition to EBNA-1 and the non-coding RNAs mentioned above (latency II). LMP1 induces tumors in transgenic animals [53,54] as a co-carcinogen [55] and has transforming potential in tissue culture [56]. LMP1 mimics the CD40 molecule [57]. LMP2A blocks the B cell receptor (BCR) [58] and can functionally replace BCR [59]. Both LMP1 and LMP2A can activate cellular signaling pathways such as the PI3K/Akt, NF-κB, Wnt and JAK/STAT pathways [60,61,62,63]. In a cord-blood humanized mouse model, LMP1 and LMP2A cooperate in the generation of EBV-induced B cell lymphoma [64].

Under immunosuppression after organ transplantation or HIV infection, EBV-infected B-lymphocytes may grow out. The tumor cells in the so-called post-transplant lymphoproliferative disease (PTLD) express the full set of latent proteins including the LMPs, the EBV nuclear antigens 1–6 and all non-coding RNAs, including the so-called BHRF1 miRNAs that are expressed from the 3′UTR of the BHRF1 mRNA [65] (latency III) (Table 1).

Multiple sclerosis (MS) appears to be a multi-factorial disease [66], with EBV being the indispensable trigger [67]. The infection of adolescents or adults may lead to infectious mononucleosis [68], which increases two-fold the probability for the subsequent induction of multiple sclerosis [69]. Elevated antibody titers to the EBV nuclear antigen 1 (EBNA-1) often precede the onset of MS [70]. Cross- reactive antibodies to EBNA-1 acerbate the immune reaction to glial cells [71]. Vitamin D deficiency also plays an important role in MS [72]. EBNA-1 binds CK2, which might interfere with the maintenance of vitamin D levels in the infected cells. Elevated levels of CK2 are not only found in many types of cancer but may function in reducing the levels of the cancer-preventing vitamin D [73].

The infection with EBV, at least as a cofactor in the induction and possibly the maintenance in the various tumors, may be assumed: nasopharyngeal carcinoma has a strong geographic and viral (EBV) component as virtually all undifferentiated NPC are EBV positive [74]. The presence of EBV in various tumors of B- and T-cells as well as ones of epithelial origin has been established [75]. First detected in endemic Burkitt’s lymphoma, EBV was subsequently found in various lymphoma such as Hodgkin’s disease (HD), diffuse large B-cell lymphoma (DLBCL), and in virtually all cases of nasal/NK T-cell lymphoma (NKTL) [76]. The virus is also present in about 15% of gastric carcinoma (GC) cases, which are as NPC of epithelial origin [75].

In vitro, EBV readily transforms resting B-lymphocytes into permanently growing cell lines (LCLs), which express the full complement of so-called “latent” genes [77], including the BART and the BHRF1 miRNAs. These lymphoblastoid cell lines (LCLs) are the in vitro complement of the PTLDs that occur in immune-compromised patients. Depending on the type of tumor, different latent genes are expressed. In Burkitt’s lymphoma (BL, latency I), EBNA-1 is the only detectable EBV protein in addition to the non-(protein)-coding EBER RNAs and the BART microRNAs. A possible direct role in tumorigenesis has been suggested by the induction of EBNA-1-bearing tumors in transgenic mice [78], a supposition that was challenged, however, by a subsequent report that used the same mouse strain [79]. The EBER transcripts are present in all EBV-positive tumors and inhibit interferon-α-mediated apoptosis, possibly via the phosphorylation of eukaryotic initiation factor α (eIF2α) by PKR [80]. The proposition that interferon synthesis was inhibited via EBER/PKR was, however, subsequently challenged [81]. EBV encodes 44 miRNAs (http://www.mirbase.org/index.shtml, accessed on 2 November 2022), only five of which are present in the fully transforming B95.8 strain [82]. Deletion of the three BHRF1 miRNAs from B95.8 results in a virus with a 20-fold reduction in transformation capacity [83]. The EBV-encoded miRNAs target genes that are implicated in proliferation, apoptosis and cell transformation and in targeting viral gene products in order to escape from the cellular immune response [84].

The EBV early protein EB-2, also known as BMLF1, Mta or SM protein, is expressed in the initial phase of lytic replication [85,86].

## 3. Phosphorylation of EBV Proteins by Protein Kinase CK2

Epstein–Barr nuclear antigen 1 (EBNA-1) is required for the replication of the EBV genome as an extra-chromosomal element and is a key transcriptional regulator of EBV latent gene expression [87]. EBNA-1 is indispensable for the immortalization of B-lymphocytes and is present in all EBV associated tumors [88]. The EBNA-1-mediated disruption of PML bodies is an important factor for the development of gastric cancer [89] and nasopharyngeal carcinoma (NPC) [90]. Through affinity chromatography and tandem affinity purification (TAP), each CK2 subunit was found to associate with EBNA-1 [91]. Sivachandran et al. showed that EBNA-1 binds to the CK2β subunit and CK2α appears to be tethered via CK2β to EBNA-1 [92]. A KSSR motif on the polypeptide chain of CK2β near the CK2β dimerization domain represents the interacting sequence for the binding to EBNA-1 [93]. EBNA-1 is a phosphoprotein [94,95]. It contains at least three putative CK2 phosphorylation sites. To our knowledge, CK2 phosphorylation of EBNA-1 has not been shown so far. One might speculate that EBNA-1 targets CK2 to other cellular proteins to induce their phosphorylation.

One of the target proteins appears to be the promyelocytic leukemia nuclear bodies (PML-NB). EBNA-1 disrupts the tumor-suppressive PML-NB, leading to an impaired DNA repair and an increased cell survival [96]. EBNA-1 and in particular its association with CK2, is necessary for the disruption of PML-NB by degradation of the PML proteins upon phosphorylation [97,98]. CK2 phosphorylates PML at serine 517, which leads to its polyubiquitylation and degradation [97,98]. Phosphorylation of EBNA-1 at the CDK phosphorylation site serine 393 [99] is critical for the interaction of EBNA-1 with PML proteins as well as for their degradation [93]. EBNA-1 mutants, which are defective for the binding of CK2 had a decreased ability to induce PML degradation.

EBNA1 is essential for the maintenance of the viral episome. In the dividing tumor cell, the viral DNA is replicated synchronously with the cellular DNA and evenly distributed to the daughter cells via EBNA-1, which tethers the viral DNA to the mitotic cellular DNA. Phosphorylation of serine(s) next to a stretch of methylated arginines within an arginine–glycine (RG) repeat is important for the segregation of the viral genome to the daughter cells during mitosis [100].

In 1992, EBNA-2 was identified as a substrate for CK2 and the phosphorylation sites were found to be serine 469 and serine 470 [101] (Table 2). EBNA-2 binds to the heterogeneous ribonucleoprotein –K (hnRNP-K) and this binding leads to an enhanced expression of LMP2A [102]. Interestingly, hnRNP-K also interacts with CK2β and the immediate-early protein 2 of human herpes virus 6 (HHV-6) [103]. Herpes virus-1 (HSV-1) infection stimulates the CK2 activity and the redistribution of CK2 from the nucleus to the cytoplasm. Furthermore, CK2 is complexed with the immediate-early protein IE63, also known as ICP27, of HSV-1. Likewise, CK2 binds and phosphorylates hnRNP-K [44,104,105]. It would be an interesting question whether EBV infection would also stimulate CK2 kinase activity, cytoplasmic localization and phosphorylation of hnRNP-K. The RG-repeat of EBNA-2 confers binding to EBNA-2-regulated promoters [106,107,108]. It is therefore possible that DNA binding of both, EBNA-1 and EBNA-2, is regulated by simultaneous phosphorylation and arginine methylation.

The latent membrane protein 1 (LMP1), expressed in type II and III latency, is a transforming protein ([109] and reviewed in [110]) (Table 1). LMP1 consists of 386 amino acids. It is an integral membrane protein with a C-terminal cytoplasmic tail, which is engaged in intracellular signal transduction [111]. The C-terminus contains two trans-activating regions (CTARs), one is located between amino acids 194–232 (CTAR1), which also harbors the CK2 phosphorylation sites and which is known to activate the NF-κB signaling pathway [112] and the PI3K/Akt pathway [113]. Chi et al. reported that CK2 phosphorylates LMP1 at least in vitro [92] (Table 2). Using bacterially expressed fragments of LMP1 revealed that the C-terminus harbors CK2 phosphorylation site(s). Later, serine residues at position 211 and 215 of LMP1 were determined as substrates for CK2 in vitro [114]; at least serine 215 of LMP1 was also found to be phosphorylated in human cell lines. The observation that serine 211 is only phosphorylated in the presence of phospho-serine 215 points to a hierarchical phosphorylation, which was reported for other proteins by Litchfield and co-workers [115]. The functional consequence of the CK2 phosphorylation of LMP1, however, remains to be elucidated.

The ZEBRA protein, also known as EB-1, Zta or BZLF1, is another EBV-encoded protein which plays a role in the disruption of viral latency and the initiation of the viral lytic cycle [46]. ZEBRA, a member of the bZIP family, binds to DNA to initiate viral replication where it functions as a transcription factor. It is a multifunctional protein that also binds to cellular and viral proteins. Serine 167 and serine 173 were mapped as in vivo and in vitro CK2 phosphorylation sites [116,117] (Table 2). By mutating the CK2 phosphorylation sites into alanine and also through the inhibition of the CK2 kinase activity, it was shown that the CK2 phosphorylation of ZEBRA leads to impaired DNA binding activity [116,118].

The EBV early protein 2 (EB-2, also known as BMLF1, Mta or SM) is responsible for the nuclear export of a subset of early and late viral mRNAs and for the production of infectious viruses [119]. EB-2 was detected as a phosphoprotein in EBV-infected cells. It can be phosphorylated by CK2 at least in vitro [120]. Furthermore, MALDI-TOF analysis and co-immunoprecipitation experiments showed that CK2α and CK2β subunits co-purify with EB-2. Mutant analysis with EB-2, where the CK2 phosphorylation sites were replaced by non-phosphorylatable alanine residues, revealed that the CK2 phosphorylation of at least one of the serine residues 55, 56 and 57 of EB-2 is critical for the production of infectious virus [121,122] (Table 2).

**Table 2:** CK2 phosphorylation of EBV proteins.

Protein	Phosphorylated Amino Acid	Reference
EBNA-2	469, 470	[101]
LMP1	211, 215	[92,114]
EB-1 (ZEBRA)	167, 173	[116,117]
EB-2 (SM)	55, 56, 57	[121]

## 4. CK2 and Cellular Proteins in the Balance between Lytic EBV Virus Replication and Cell Transformation

### 4.1. Ikaros and the Switch from EBV Latency to Lytic Replication

Ikaros is a zinc finger, DNA-binding transcriptional regulator [123]. Functions of Ikaros are regulated by post-translational modifications such as phosphorylation and sumoylation [124,125,126,127]. CK2 phosphorylates Ikaros at multiple sites [128,129,130]. In particular, the N-terminal CK2 phosphorylation of Ikaros reduces its DNA binding affinity and thereby its transcription factor activity [131]. The role of CK2 in the regulation of the transcriptional activity of Ikaros has very recently been reviewed by Bogush et al. [132]. It was shown that Ikaros plays a role in the maintenance of viral latency in EBV-positive Burkitt’s lymphoma [133]. So far it has not been directly shown, but it is tempting to speculate, that the CK2 phosphorylation of Ikaros might play a role in the Ikaros-mediated switch from latency to lytic replication of EBV.

### 4.2. CK2 Binding Cellular Protein ARKL1 and the Regulation of EBV Replication

EBV maintains a life-long infection in humans through a switch between a latent and a lytic replication cycle [68]. The ARKADIA-like-1 (ARKL1) protein acts as a negative regulator of EBV reactivation for a lytic infection by interacting with c-jun. The silencing of CK2β abrogates the ARKL1 c-jun interaction and thereby EBV reactivation [134]. This function is explained by the fact that EBNA-1 binds to the same KSSR motif in the polypeptide chain of CK2β rather than ARKL1 [93]. There is increasing evidence that ARKL1 has a more general anti-viral function because it was shown that it also inhibits influenza virus infection [135] and human T-cell leukemia virus type 1 (HTLV-1) infection [136].

### 4.3. CK2 and the Autoregulatory Loop between NF-κB, BARTs and LMP1

**Figure 1:**
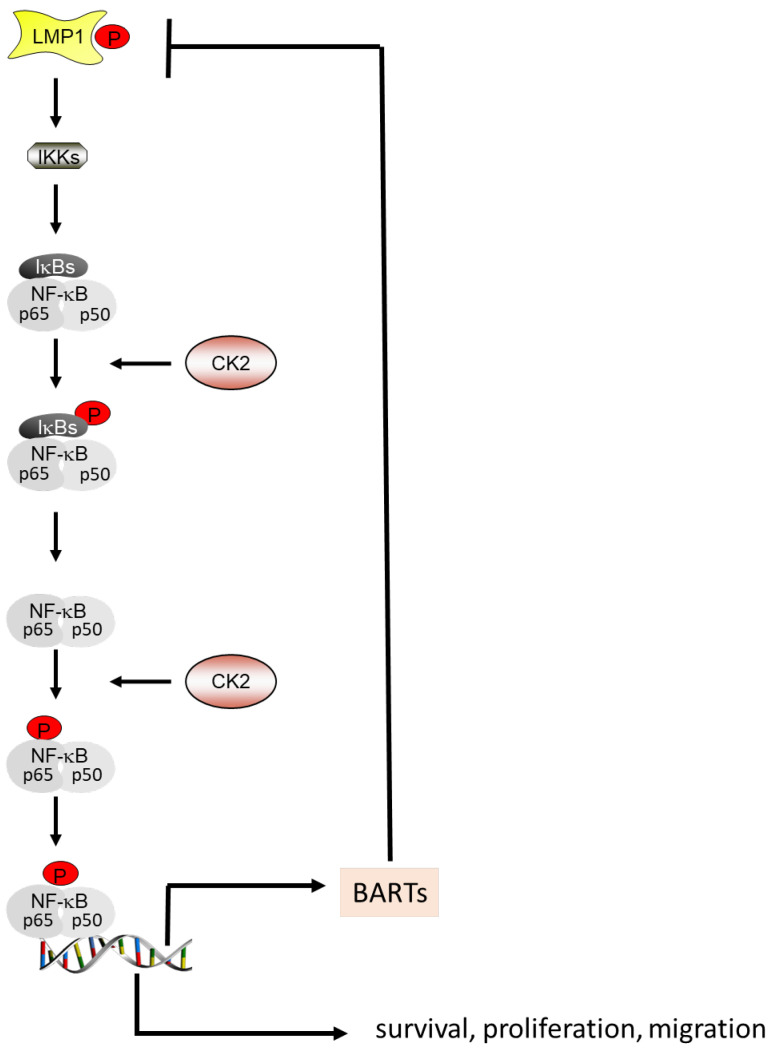
LMP1, BARTs, CK2 and the NF-κB signaling pathway. LMP1, NF-κB and BARTs are implicated in an autoregulatory loop. CK2 phosphorylates and thereby stimulates the NF-κB signaling pathway. As an activated transcriptional regulator, NF-κB increases the expression of BARTs and the expression of genes implicated in cell survival, proliferation and migration.

BARTs are expressed in all types of EBV-infected cells and in EBV-associated tumors [137,138]. High levels of BARTs are associated with the maintenance of the oncogenic state of NPC. The NF-κB family of transcription factors plays an essential role in inflammation and cancer initiation and progression [139]. Several members of the NF-κB activation cascade are phosphorylated by CK2 and thereby activated for transactivation [140]. NF-κB regulates the expression of BARTs, which repress LMP1 expression. Furthermore, LMP1 is implicated in the activation of the NF-κB signaling cascade. This autoregulatory loop seems to regulate the balance between lytic proliferation and cell transformation [141] (Figure 1).

## 5. Common Targets of EBV and CK2 

### 5.1. EBV, CK2 and the NF-κB Pathway

EBV is thought to exert its oncogenic potential via different cellular signaling pathways, including the NF-κB signaling pathway [142]. LMP1 enhances the NF-κB signaling and activates NF-κB transcription factor activity [65]. NF-κB is a transcription factor complex consisting of p50, p52, RelAp65, RelB and RelC subunits. For the immortalization of EBV-infected cells, LMP1 activates RelAp65 to bind to the human telomerase reverse transcriptase (hTERT) to activate telomerase and thereby induce EBV-mediated immortalization. It has been known for quite some time that CK2 regulates the NF-κB pathway through the phosphorylation of several components of this pathway, such as RelAp65, IκB, IKK2 and NF-κB [143,144,145,146,147] (Figure 1). In the nucleus, CK2 binds to and phosphorylates RelAp65 at serine 529 [148]. The CK2 phosphorylation of IκB promotes its degradation and thereby NF-κB activation. In vitro phosphorylation experiments have shown that CK2 phosphorylates IKK2. The inhibition of CK2 kinase activity with apigenin- or siRNA-targeting CK2β completely inhibited IKK2 phosphorylation [149] and NF-κB activity. The incubation of IKK2 with recombinant CK2α leads to an increased activity of IKK2 for the phosphorylation of serine 32 and serine 36 in the N-terminus of IKBα. On the other hand, CK2 promotes the IKK-mediated activation of NF-κB [149]. As mentioned above, LMP1 is phosphorylated by CK2 [92,114]. It remains, however, to be elucidated whether the CK2 phosphorylation of LMP1 is necessary for the stimulation of the NF-κB signaling cascade (Figure 1).

### 5.2. EBV, CK2 and the PI3K/Akt Pathway

**Figure 2:**
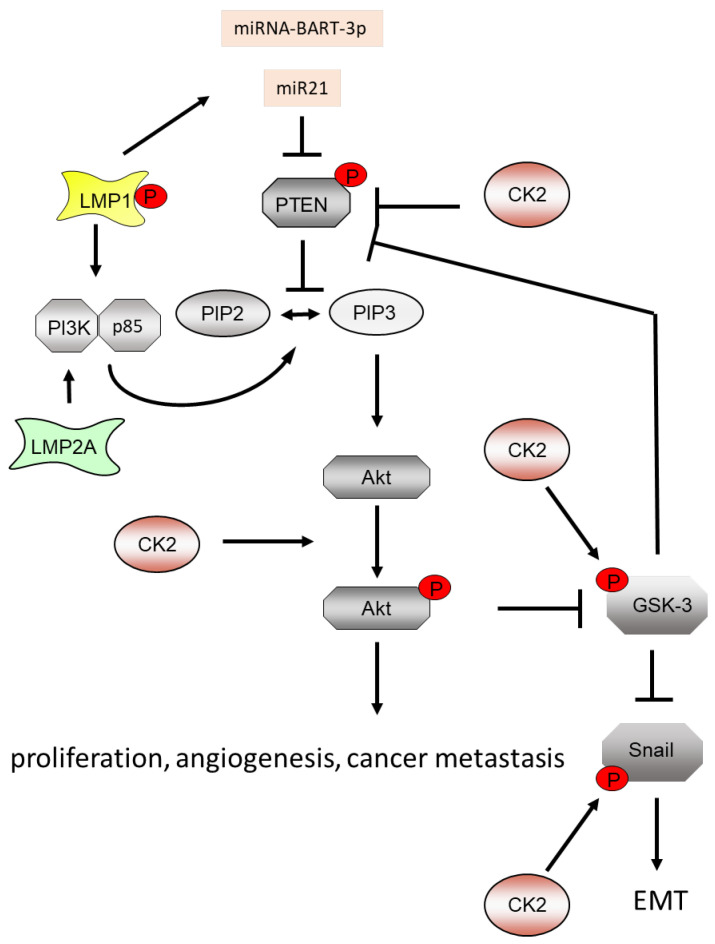
LMP1, miRNA-BART-3p and CK2 regulate the PI3K/Akt signaling pathway. CK2 phosphorylates PTEN, Akt and Snail to regulate the PI3K/Akt signaling pathway in order to induce proliferation, angiogenesis, cancer metastasis and epithelial and mesenchymal transition (EMT).

Another signaling pathway that is implicated in EBV-mediated cell transformation is the PI3K/Akt signaling pathway (Figure 2). LMP1 and LMP2A activate PI3K [150,151]. The PI3K/Akt signaling pathway plays a major role in cancer development [152]. PI3K is upstream regulated by the phosphatase and tensin homologue (PTEN) and regulates downstream Akt kinase, also known as protein kinase B (PKB). PTEN converts phosphatidylinositol-3,4,5-triphosphate (PIP3) into phosphatidylinositol-4,5-bisphosphate (PIP2). CK2 phosphorylates PTEN at serine 370, serine 380, threonine 382, threonine 383 and serine 385, which results in an increase in PTEN protein stability [153]. Miller et al. found that serine 370 and serine 385 are the main CK2 phosphorylation sites, which are responsible for the inhibition of the phosphatase activity of PTEN for its substrate PIP3 and the inhibition of the caspase-3 cleavage of PTEN [153,154]. Thus, CK2 phosphorylation of PTEN leads to an elevated protein stability of PTEN and PTEN inactivation [155]. The inhibition of CK2 by CX-4945 reverses PTEN stabilization, which leads to an elevated cell death by an inhibition of the PI3K/Akt pathway. CK2 phosphorylates GSK3β, which also phosphorylates PTEN [156] in a cooperative way; i.e., CK2 phosphorylation at serine 370 strongly enhances the subsequent phosphorylation at threonine 366 by GSK3β [157]. The threonine 366 phosphorylation leads to destabilization of PTEN. However, this appears to be a cell type specificity of the phosphorylation events [157,158]. Akt phosphorylates GSK3β and this results in an inhibition of GSK3β (Figure 2).

The EBV-mi-Bart7-3p is highly expressed in nasopharyngeal carcinoma (NPC) and positively correlated with lymph node metastasis and the clinical stage of NPC [159]. miR-Bart7-3p promotes the transition from the epithelial to the mesenchymal phenotype by regulating PTEN/PI3K/Akt, GSK2β, Snail and β-catenin. Snail is tightly regulated at the transcriptional and post-transcriptional levels. The GSK3β phosphorylation of Snail regulates Snail protein stability and nuclear export [160]. Furthermore, CK2 in vitro and in vivo phosphorylates Snail at serine 92. By using a yeast two hybrid screen, pull-down assays and co-immunoprecipitation analysis, CK2 was identified as a binding partner of Snail. By replacing serine against the non-phosphorylatable alanine, it was shown that the CK2 phosphorylation at serine 92 of Snail is required for the efficient transcriptional repression of E-cadherin. Furthermore, serine 92 phosphorylation appears to increase Snail degradation [160] (Figure 2).

Furthermore, LMP1 downregulates the expression of PTEN by enhancing the expression of miR-21, thereby activating the PI3K/Akt pathway [161]. LMP1 also activates the PI3K/Akt pathway and the HIF1α signaling in EBV positive nasopharyngeal carcinomas (NPCs) facilitating vascularization of the tumor [162]. LMP1 interacts with the p85 subunit of PI3K, which leads to an activation of src. Src enhances the activity of the interferon regulatory factor 4 (IRF4) and thereby promotes cell transformation [163].

### 5.3. EBV, CK2 and the Wnt/β-Catenin Pathway

**Figure 3:**
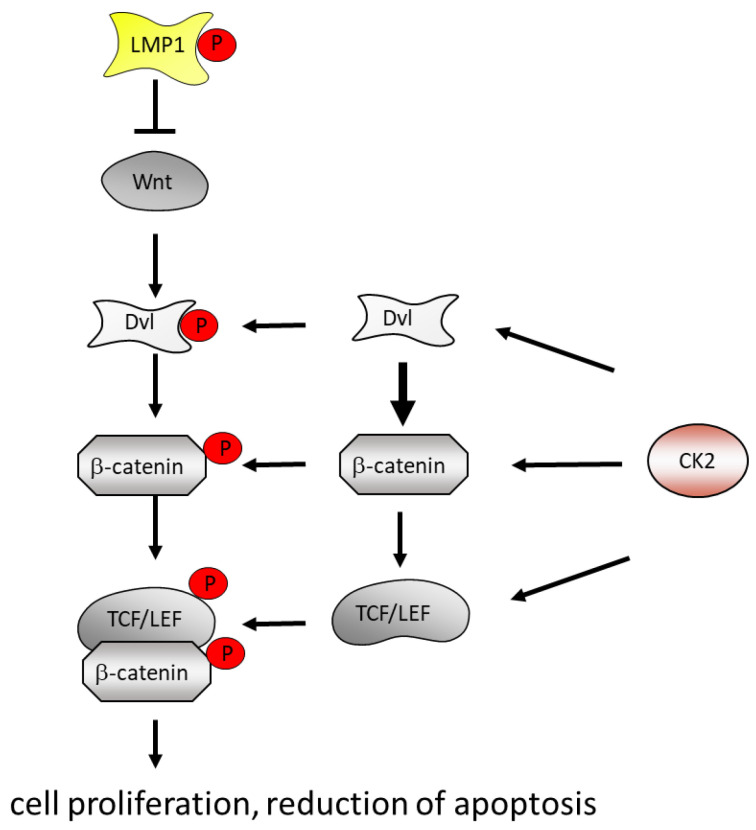
LMP1 and CK2 cooperate in the activation of the Wnt, Dishevelled and β-catenin pathway. CK2 phosphorylates and activates Wnt, Dishevelled and β-catenin to promote cell proliferation and a reduction in apoptosis.

Wnt signaling is another pathway which is implicated in the EBV-induced cell transformation [164] (Figure 3). Activation of the Wnt signaling pathway leads to an increase in cell survival and a reduction in apoptosis [85]. CK2 is implicated in Wnt signaling through its association with and its phosphorylation of Dishevelled (Dvl), which is in a multi-protein complex containing β-catenin. CK2 phosphorylates β-catenin and thereby promotes its stability and translocation into the nucleus [165,166]. The inhibition of CK2 by TBBt leads to an even intracellular distribution of Dishevelled and inhibits a further phosphorylation by CK1ε and thereby an activation of TCF/LEF-mediated transcription [167] (Figure 3). Moreover, CK2 phosphorylates Akt at serine 129 [18,168,169]. This CK2 phosphorylation appears not to be an on or off signal but to increase the activity of the Akt kinase. CK2 phosphorylation at serine 129 hyperactivates Akt for the phosphorylation of β-catenin at serine 552, which promotes its nuclear accumulation and transcriptional activation. In addition, CK2 itself phosphorylates β-catenin at threonine 393, which protects β-catenin from proteasome-dependent degradation and increases its transcriptional activity [165].

### 5.4. EBV, CK2 and the Janus Kinase/Signaling Transduction and Transcription Activator (JAK/STAT) Pathway

**Figure 4:**
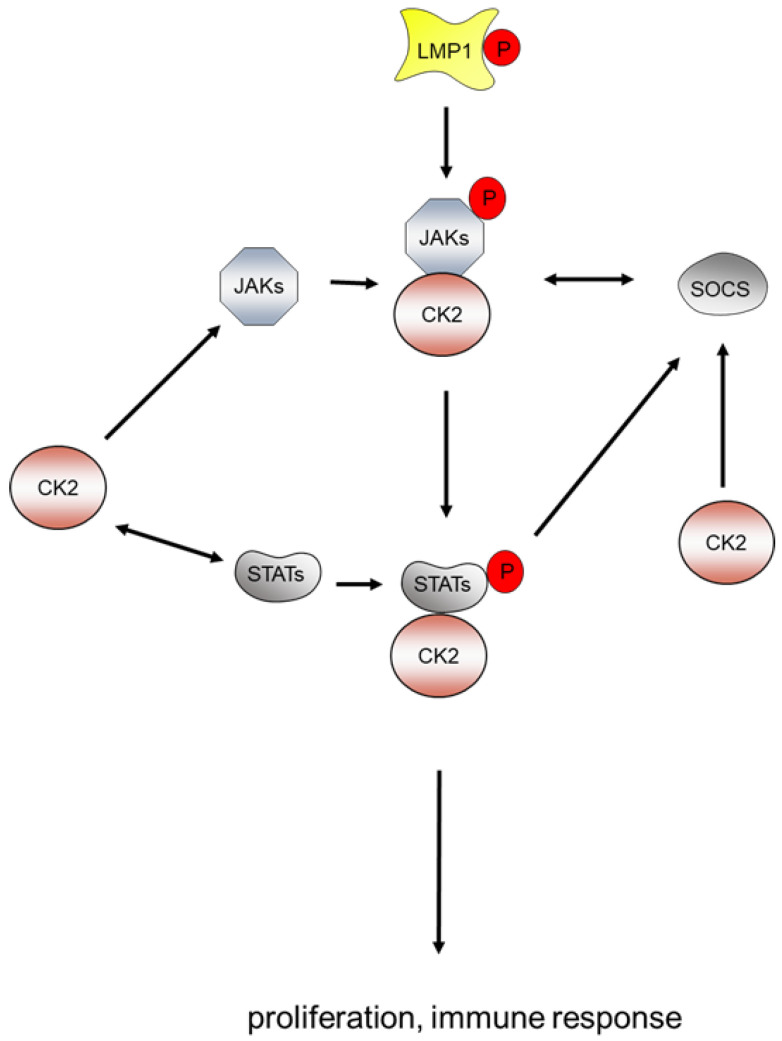
LMP1 and CK2 cooperate in the activation of the JASK/STAT signaling pathway. CK2 binds to JAK and STAT and phosphorylates both proteins to promote proliferation and the immune response.

The tyrosine kinases of the JAK family are either activated by growth factors and cytokines or as the result of mutations [170]. In EBV-positive diffuse large B-cell lymphoma (DLBCL), there is some indication that the JAK/STAT pathway is activated [171]. LMP1 appears to trigger the JAK/STAT pathway by the regulation of the JAK3 expression as well as the phosphorylation of STAT [172]. CK2 is an interaction partner of the JAKs and essential for the activation of the JAK/STAT pathway [170]. CK2 phosphorylates STAT1 [173] and STAT3 [174] and also JAK2 and JAK3 [170], which results in an amplification of cytokine signals [170,175]. CK2 itself is under the control of STAT3 [176], which might indicate an auto-regulatory loop. Co-immunoprecipitation experiments have revealed that CK2α and CK2β bind to JAK1 and JAK2. The expression of cytokine signaling 3 (SOCS-3) is inhibited by siRNA technology targeting CK2 or by pharmacological inhibition of the enzyme activity of CK2 [170] (Figure 4).

## 6. EBV-Encoded LMP1 Protein and p53

p53 is a tumor suppressor which regulates the eukaryotic cell cycle and apoptosis (for review see: [177]). Since viruses depend on host cells for their replication, the guardian of the genome p53 [178] plays a central role in the host defense against a virus infection [179]. It was shown that an EBV infection interferes with cell cycle checkpoint control [180,181] and affects p53 stability [180,182]. There was a controversy whether LMP1 represses DNA repair by p53 and inactivated the transcriptional activity of p53 [183] or whether it activates p53 transcriptional activity and increases the stability of p53 through multi-sites phosphorylation [184,185]. In another study, it was shown that the overexpression of LMP1 led to a poly-ubiquitination of p53 followed by a decrease in p53 levels [186]. p53 is phosphorylated by different protein kinases, including CK2, and it is associated with CK2 [187,188,189,190]. The phosphorylation of p53 by CK2 at serine 392 leads to the stabilization of p53 protein [191], indicating that this phosphorylation might counteract LMP1 activity.

## 7. Conclusions

In the present review, we demonstrated that protein kinase CK2 is strongly implicated in the regulation of EBV viral replication, in persistent infection and in cell transformation leading to cancer. These different functions are achieved through the phosphorylation of virally encoded proteins as well as through the phosphorylation of cellular proteins, which are regulators of cellular signaling pathways such as the NF-κB, PIP3/Akt, JAK/STAT, and Wnt/Dishevelled/β-catenin signaling pathways. CK2 and EBV act on the same cellular signaling pathways. It remains to be elucidated whether and how EBV hijacks CK2 to influence these different signaling pathways for neoplastic transformation. The binding of CK2 subunits to viral and cellular proteins might reflect an enzyme–substrate interaction. Alternatively, the interactions might target CK2 to other substrates. Since a great number of different CK2 kinase inhibitors are now known, some of which have already been used to inhibit signaling pathways, these inhibitors are promising tools for the inhibition of virus replication as well as of virally induced cancers. Very recently, CK2 was found to play a role in SARS-CoV-2 infections [45]. The knowledge of the role of CK2 in EBV infection might also help to find new strategies to fight COVID-19 [192].

## Figures and Tables

**Table 1:** Latency type and expression of EBV transcripts.

Gene(s)/Protein(s) Expressed in EBV Latency Patterns								
Latency Type	EBERs	BART miRNAs	EBNA1	LMP1/2	EBNA2-6	BHRF1 BHRF1-miRNAs	v-snoRNA	Cell Type/ Tumor Type
0	+	+						Memory B-cell
I	+	+	+					BL/GC B-cell
II	+	+	+	+				HD/NPC/DLBCL
III	+	+	+	+	+	+	+	PTLD/LCLs

Abbrevations: BL: Burkitt’s lymphoma; GC: germinal center; HD: Hodgkin’s disease; NPC: nasopharyngeal carcinoma; DLBCL: diffuse large B-cell lymphoma; BART: BamHI rightward transcript; BHRF1: BamHI rightward open reading frame 1; PTLD: post-transplant lymphoproliferative disease; LCL: lympoblastoid cell line.

## Data Availability

Not applicable.

## Glossary

BARF1	BamHI-A rightward open reading frame 1
BARTs	BamHI-A rightward transcripts
BZLF1 or ZEBRA or EB-1 or Zta	BamHI-Z EBV replication activator
BL	Burkitt’s lymphoma
DLBCL	Diffuse large B-cell lymphoma
PKR	Double-stranded RNA-dependent protein kinase
EBER	Epstein–Barr encoded small RNA
EBV	Epstein–Barr virus
EBNA	Epstein–Barr virus nuclear antigen
GC	Gastric carcinoma
HCV	Hepatitis C virus
HSV	Herpes simplex-1 virus
HD	Hodgkin’s disease
HCMV	Human cytomegalovirus
HIV	Human immunodeficiency virus
HTLV-1	Human T-lymphotropic virus type 1
JAK	Janus kinase
LMP	Latent membrane protein
MS	Multiple sclerosis
NKTL	Nasal NK/T-cell lymphoma
NPC	Nasopharyngeal carcinoma
NF-kB	Nuclear factor kappaB
PI3K	Phosphatidylinositol-3-kinase
PTLD	Post-transplant lymphoproliferative disease
Akt	Protein kinase B, also known as Akt
CK2	Protein kinase CK2
PKC	protein kinase C
SARS-CoV-2	Severe acute respiratory syndrome coronavirus type-2
STAT	Signal transducer and activator of transcription protein
